# Study protocol of transcranial electrical stimulation at alpha frequency applied during rehabilitation: A randomized controlled trial in chronic stroke patients with visuospatial neglect

**DOI:** 10.1186/s12883-022-02932-7

**Published:** 2022-11-02

**Authors:** Marij Middag-van Spanje, Teresa Schuhmann, Tanja Nijboer, Olof van der Werf, Alexander T. Sack, Caroline van Heugten

**Affiliations:** 1 Section Brain Stimulation and Cognition, Department of Cognitive Neuroscience, Faculty of Psychology and Neuroscience, Maastricht University, Maastricht, The Netherlands; 2 InteraktContour, Nunspeet, The Netherlands; 3 Maastricht Brain Imaging Centre, Maastricht University, Maastricht, The Netherlands; 4 Department of Experimental Psychology, Helmholtz Institute, Utrecht University, Utrecht, The Netherlands; 5 Center of Excellence for Rehabilitation Medicine, UMC Utrecht Brain Center, University Medical Center Utrecht and De Hoogstraat Rehabilitation, Utrecht, The Netherlands; 6 Centre for Integrative Neuroscience, Faculty of Psychology and Neuroscience, Faculty of Health, Medicine and Life Sciences, Maastricht University, Maastricht, The Netherlands; 7 Limburg Brain Injury Center, Maastricht, The Netherlands; 8 Department of Neuropsychology & Psychopharmacology, Faculty of Psychology and Neuroscience, Maastricht University, Maastricht, The Netherlands; 9 School for Mental Health and Neuroscience, Department of Psychiatry & Neuropsychology, Faculty of Health, Medicine and Life Sciences, Maastricht University Medical Center, Maastricht, The Netherlands

**Keywords:** Stroke, Visuospatial neglect, Visual scanning training, Transcranial electrical stimulation, Transcranial alternating current stimulation, Randomized controlled trial, Activities of daily living

## Abstract

**Background:**

A frequent post stroke disorder in lateralized attention is visuospatial neglect (VSN). As VSN has a strong negative impact on recovery in general and independence during daily life, optimal treatment is deemed urgent. Next to traditional stroke treatment, non-invasive brain stimulation offers the potential to facilitate stroke recovery as a complementary approach. In the present study, visual scanning training (VST; the current conventional treatment) will be combined with transcranial alternating current stimulation (tACS) to evaluate the additive effects of repeated sessions of tACS in combination with six-weeks VST rehabilitation.

**Methods:**

In this double-blind randomized placebo-controlled intervention study (RCT), we will compare the effects of active tACS plus VST to sham (placebo) tACS plus VST, both encompassing 18 VST training sessions, 40 minutes each, during 6 weeks. Chronic stroke patients with VSN (> 6 months post-stroke onset) are considered eligible for study participation. In total 22 patients are needed for the study. The primary outcome is change in performance on a cancellation task. Secondary outcomes are changes in performance on a visual detection task, two line bisection tasks, and three measures to assess changes in activities of daily living. Assessment is at baseline, directly after the first and ninth training session, after the last training session (post training), and 1 week and 3 months after termination of the training (follow-up).

**Discussion:**

If effective, a tACS-VST rehabilitation program could be implemented as a treatment option for VSN.

**Trial registration:**

ClinicalTrials.gov; registration number: NCT05466487; registration date: July 18, 2022 retrospectively registered; https://clinicaltrials.gov/ct2/show/NCT05466487

## Background

Visuospatial neglect (VSN) is a common syndrome after unilateral stroke; 25-30% of all stroke patients have VSN [1, 2]. VSN patients show a failure or slowness to report, respond or orient to events and stimuli located in the contralesional side of space [2, 3]. It is thought that the brain damage causes impairment of the brain’s spatial attention mechanisms, resulting in VSN [4, 5]. VSN patients show slower and more attenuated motor recovery patterns [6] and need more help in activities of daily living (ADL) compared to stroke patients without VSN [7]. Moreover, VSN negatively influences participation in society, increases caregiver burden [8] and is negatively related to life satisfaction [9]. These findings show the considerable impact of VSN on daily life and stress the importance of adequate treatment.

Over the past decades, many therapeutic interventions aiming to improve VSN have been developed and evaluated (see for overview [10]) ranging from treatments using top-down strategies such as mental imagery training [11], to bottom-up methods such as prism adaptation [12, 13], and from sustained attention training [14], to non-invasive brain stimulation techniques (NIBS) [15]. Currently, the standard treatment for VSN is a visual scanning training (VST), an intensive compensatory training with emphasis on top-down strategies designed to improve viewing and searching behavior [16]. However, generalization of the effects of VST to everyday life is insufficiently established [10, 17] and there exists a large variability in patients’ benefits from VST [18]. It is unclear why some patients benefit from the training while others do not. One reason could be that top-down methods such as VST may be limited as they solicit the attentional abilities, which may be hampered by lack of awareness of the spatial neglect behavior [10, 18]. The heterogeneity of VSN, with high variability of symptoms within and between patients, may also play a role in the variability of responsiveness to interventions for neglect [12]. Because neglect is a multifaceted disorder, it is suggested that the best treatment might involve a combination of different methods to improve their overall effectiveness [10, 12, 15, 19–21].

NIBS offers a completely different strategy to facilitate recovery, not by means of a behavioral approach submitting the patient to a program of standardized behavioral tasks that require a voluntary (attentional) effort by the patient to follow a therapist’s instructions (such as VST), but by directly inducing neuroplastic changes in the patient’s brain, hoping to positively affect cognitive functioning. For example, brain stimulation protocols can be tuned to modulate oscillatory brain activity (for review see [22]). This is particularly interesting in the field of neglect rehabilitation, as oscillatory activity in the alpha range (8-12 Hz) in posterior parietal cortices has been linked to spatial attention bias in healthy subjects [23–27]. In our recent studies, we showed that transcranial alternating current stimulation (tACS), applied at alpha frequency, can be used to influence visuospatial attention performance in healthy participants [28] and in sub-acute VSN patients [ 29] in a single session.

To our knowledge, no study has yet reported the combined impact of this oscillatory-based NIBS approach and conventional neglect therapy on rehabilitation outcome. The overall aim of the current study is therefore to evaluate the effects of repeated sessions of tACS in combination with six-weeks VST rehabilitation. Our primary research question is: Does VST complemented with active tACS improve neglect-related symptoms to a larger extent compared to VST with sham (placebo) tACS post training compared to baseline? Secondary questions are: 1) whether long-lasting effects occur, 2) whether effects already occur earlier during the six-weeks training, and 3) whether effects generalize to daily-life situations.

## Methods

### Design

**Fig. 1:**
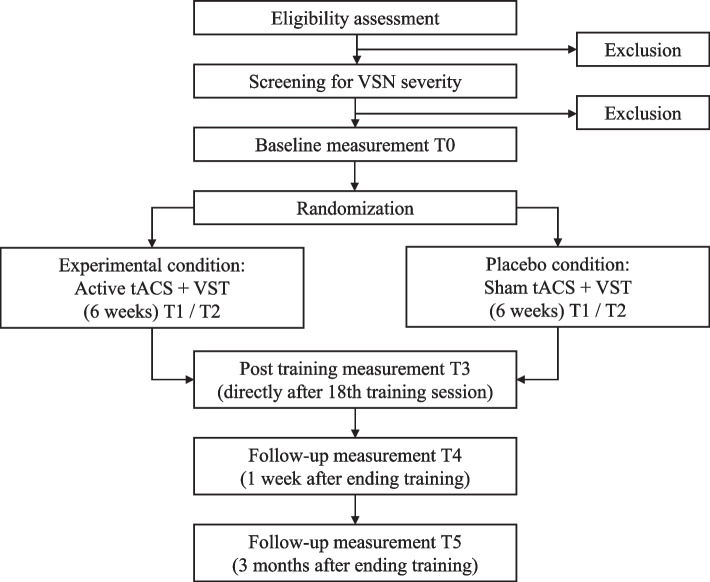
Study flow chart

This study is a double-blind randomized placebo-controlled intervention study (RCT; Fig. 1). We will compare the effects of active tACS to sham (control) tACS, both combined with conventional rehabilitation (VST). Irrespective of the intervention group, *all* patients will receive VST during the (active or sham) stimulation.

This study is conducted according to the principles of the Declaration of Helsinki (59th WMA General Assembly, Seoul, Korea, October 2008) and in accordance with the Medical Research Involving Human Subjects Act (WMO). The study is approved by the Medical-Ethical Committee azM/UM of Maastricht University (NL70256.068.19 / METC 19-047) and registered in ClinicalTrials.gov (Non-Invasive Brain Stimulation as an Innovative Treatment for Chronic Neglect Patients (NibsNeglect), NCT05466487).

### Patient sample – inclusion and exclusion criteria

Patients with a clinically diagnosed, chronic stroke and with signs of neglect symptoms (based on clinical judgment), will be considered eligible for our study. Patients will be recruited by psychologists of healthcare organizations in The Netherlands that are specialized in supporting and treating people with acquired brain injury (InteraktContour, De Hoogstraat Revalidatie, Heliomare, De Noorderbrug, Esdégé-Reigersdaal). The inclusion of participants started in September 2020 and data will be collected until September 2023.

Inclusion criteria are: 1) neurologically objectified stroke (first or recurrent, ischemic or intracerebral or subarachnoid hemorrhagic lesion; 2) stroke occurred when patient was 18-80 years of age; 3) chronic stroke (> 6 months post-stroke onset); 4) sufficient comprehension and communication skills to benefit from training (based on clinical judgement); and lastly 5) a screening containing four neuropsychological tests will be performed to evaluate the current severity of the neglect, since the diagnosis of neglect may have been established months or even years ago in our sample of chronic stroke patients.

Exclusion criteria are: 1) currently engaging in cognitive rehabilitation treatment or neglect treatment; 2) physically or mentally unable to participate (based on clinical judgment); 3) hemianopsia (based on clinical judgement); 4) severe communicative disability, as task descriptions need to be understood; 5) local scalp injuries; 6) eczema on scalp or psoriasis; 7) diagnosed (neuro) psychiatric or neurodegenerative diseases; 8) current alcohol and/or drug abuse; and 9) pregnancy, due to tACS safety considerations (5-9).

### Procedure, neglect screening, outcome measures, and baseline descriptors

Patients are allowed to proceed in the study when they show neglect on minimally one of the four screening tasks, on the basis of standard norms: bells task (BT) [30], balloons-subtest B (BB) [31], Schenkenberg line bisection task (SLBT) [32], and McIntosh line bisection task (MLBT) [33, 34]. Patients who show neglect during screening will be randomly assigned to either the experimental (active tACS) or placebo (sham tACS) condition, and will receive VST training for six weeks. Enrolled patients will be tested six times on an array of tasks: before the training (T0; baseline), after the first (T1), ninth (T2), and eighteenth (T3) training session, as well as one week (T4) and three months (T5) after termination of the training (Fig. 1). The star cancellation task (SCT), computerized visual detection task (CVDT), MLBT-digitized (MLBT-d), and SLBT will be assessed during all six testing-time points in the study (T0-T5). The baking tray task (BTT), Catherine Bergego scale (CBS), and subjective neglect questionnaire (SNQ) will be administered at four testing-time points (T0, T2, T4, and T5).

#### Screening tasks

##### Bells task (BT)

This cancellation task will be presented on an A4 paper and consists of 35 target items (bells), interspersed among 280 distractor objects [30]. Patients will be instructed to mark all bells. Four or more omissions are considered as indicative for VSN [18].

##### Balloons-subtest B (BB)

The scores of subtest B of the balloons test [31] will be used to calculate a total score (total number of targets cancelled) and a laterality score (number of targets cancelled on the left side of the page expressed as a percentage of the total number of targets cancelled). Subtest B consists of an A3 paper with 20 targets (circles) and 180 distractors (balloons). A total score of less than 17 *and* a laterality score of less than 45% is indicative of left VSN.

##### Schenkenberg line bisection task (SLBT)

The SLBT consists of 20 horizontal lines, varying from 10 to 20 cm in length (average 15 cm), at three different positions (left, middle, right) on a landscape-oriented A4 sheet [32]. Patients will be asked to mark their perceived midpoint of every line. The following formula will be used to calculate the relative deviation score:

$$deviation=\frac{\left( bisection\ mark- true\ center\right)}{true\ center}\cdot 100\%$$

Where values are always measured from the left end of the line. The relative deviation scores will then be averaged across all 20 lines to generate the summary score, and across the three line positions to generate the left, middle, and right average score, respectively. VSN is indicated when the average bisection mark deviates more than 10% from the true center [32].

##### McIntosh line bisection task (MLBT)

The MLBT provides a simple measure of lateral asymmetry, the endpoint weightings bias (EWB) [33, 34]. The EWB is a different approach to line bisection to diagnose VSN. Compared to classical line bisection tests, the EWB is a more theoretically neutral and parsimonious approach, based on the weight one distributes to both of the endpoints of the line, rather than using the deviation from the midpoint. The MLBT consists of 32 horizontal lines (width: 3 mm), each presented individually on an A4 sheet. The patient is asked to mark the subjective midpoint of each line. There are eight repetitions of each of four unique lines (lines A, B, C, D), presented in a fixed-random order [33, 34]. We refer to McIntosh and colleagues [33] for the arrangement of the four lines. Each response is coded as a horizontal coordinate relative to the center of the page. The analysis then focuses on how this response position varies from trial-to-trial as a consequence of changes in the left endpoint (lines A & C vs B & D) and changes in the right endpoint (lines A & B vs C & D). Thus, the left endpoint weighting (dPL), the right endpoint weighting (dPR), and the bias towards one of the two endpoints (EWB) are derived as follows:

$$dPL=\frac{\left({P}_{mean}\ \textrm{in}\ \textrm{line}\ \textrm{A}\ \textrm{and}\ \textrm{C}\right)-\left({P}_{mean}\ \textrm{in}\ \textrm{line}\ \textrm{B}\ \textrm{and}\ \textrm{D}\right)}{40}$$

$$dPR=\frac{\left({P}_{mean}\ \textrm{in}\ \textrm{line}\ \textrm{C}\ \textrm{and}\ \textrm{D}\right)-\left({P}_{mean}\ \textrm{in}\ \textrm{line}\ \textrm{A}\ \textrm{and}\ \textrm{B}\right)}{40}$$

$$EWB= dPR- dPL$$

Where dPL and dPR are expressed as a proportion of the endpoint change (40 mm), and range from 0 to 1. Perfect performance would yield symmetrical right and left endpoint weightings of 0.5, and an EWB-value of zero. An EWB-value above zero indicates a greater influence of the right endpoint (over the left), and would be a sign for left sided neglect. To define cut-off scores for the MLBT, we administered the task to healthy controls (*n* = 46, female = 47.7%, age = 57.8 years, SD = 9.2). This yielded a mean EWB of −.0217 (SD = .0546). Scores of 2 SD’s above and below the mean are considered to exceed normal range, leading to upper and lower cut-offs for left and right neglect respectively, of + 0.09 and − 0.13. Study protocol was approved by the ethical committee of the Faculty of Psychology and Neuroscience at Maastricht University (ERCPN number: 177_03_03_2017_S32).

#### Primary outcome measure

##### Star cancellation task (SCT)

The SCT is developed to detect the presence of VSN in the near extra personal space in patients with stroke, and consists of 52 large stars, 13 letters, and 10 short words interspersed with 56 smaller stars [35]. In our study, the SCT will be presented on a laptop screen (screen size: 14 in.). The patient is instructed to mark all targets by touching the screen with the finger (small stars). After each touch, a small circle appears at the touched location and remains on the screen. Two small stars in the center are used for demonstration. To determine the severity of the VSN, quality of search (QoS) for the left and right visual fields will be derived. This score combines speed and accuracy in a single measure, and is calculated using the equation as shown below [36]. A high score reflects a combination of a high number of cancelled targets, and a high cancellation speed.

$$QoS=\frac{{N_{cor}}^2}{N_{tar} \cdot {t}_{tot}}$$

Where *N*_*cor*_ is the number of cancelled targets (correct responses), *N*_*tar*_ is the total number of targets, and *t*_*tot*_ is the total time spent.

The SCT, as well as the CVDT and MLBT-d (see subsection “Secondary outcome measures”), will be administered on the same touch screen laptop as will be used for the training (HP EliteBook × 360 1040 G5 Notebook; screen size: 14 in.). PsychoPy will be used to control stimulus presentation and recording of behavioral responses.

#### Secondary outcome measures

##### Computerized visual detection task (CVDT)

The CVDT measures perceptual sensitivity and attentional selection in each hemifield [28, 29, 37, 38]. During the task, the patient is seated in front of the laptop screen at 52 cm distance. The patient is asked to fixate on the fixation cross at the center of the screen. Gabor patches (spatial frequency = 1.5 cycles per degree, envelope standard deviation = 7.5°, random orientation) are presented to the left, right and bilateral sides of the screen at 14° eccentricity. Stimuli are shown for 100 ms and stimulus size is 10°. The patient is instructed to indicate the position of the stimulus (left, bilateral, or right) by pressing the <, ˅, or > key, respectively. For each trial, the stimulus position, contrast level, and response are recorded. Video mode is 1280 × 720 at 60 Hz.

For each of the three locations (left, bilateral, right) independently, the contrast of the stimuli is adaptively changed on a trial-by-trial basis. The following parameters are used: prior grating contrast = 1, prior standard deviation = 0.5, beta = 3.5, gamma = 0.01, delta = 0.01, and aim performance = 0.5 (50% detection rate). At the beginning of the task, nine practice trails are presented (i.e. three for each condition; left, bilateral, right) for the patient to become familiar with the task. The stimuli are at maximum contrast, are not part of the staircase procedure, and are not saved. After each practice trial, short written feedback is given (‘Correct’, ‘Wrong’) in the center of the screen. Then, in the actual task, three randomly interleaved staircases are included (left, right, bilateral), with 40 trials each.

Correct hits will be weighted by the contrast level, according to the following formula:

$$weighted\ hits=\frac{\log_{10}{contrast}_{max}}{\log_{10}{contrast}_{trial}}$$

Where *contrast*_*max*_ is 100% [29]. This variable accounts for the logarithmic nature of contrast detection, and makes trials count more when the contrast was low. To illustrate, trials detected at maximum contrast receive a score of 1, whereas trials detected at minimal contrast level of 10% receive a score of 2. Performance of the CVDT will be the sum of weighted hits per condition (ipsilesional stimulus, contralesional stimulus, bilateral stimulus), resulting in a score of 0 to 76.49 per condition.

##### McIntosh line bisection task-digitized (MLBT-d)

A digitized version of the above described MLBT (subsection “Screening tasks”) is also used as study outcome measure. Each of the 32 lines of the MLBT-d are presented individually on a laptop screen. The patient is asked to mark the subjective midpoint of each line by touching the screen with the finger.

##### Schenkenberg line bisection task (SLBT)

In addition to the MLBT(−d), which is still a novel method for administering and analyzing line bisection, we will administer the SLBT, which is a simple line bisection task, widely used in the diagnosis and study of VSN [32]. A description of the SLBT is already given in subsection “Screening tasks”.

##### Baking tray task (BTT)

The patient is asked to distribute 16 cubes of 3.5 cm as evenly as possible over a 75 × 100 cm board (as if spreading out buns on a baking tray) [39]. The entire board will be scanned using the Microsoft Lens iOS app. Coordinates of all cubes will be manually identified using a custom Python script. An average positive x-coordinate indicates a rightward bias.

##### Catherine Bergego scale (CBS)

The CBS is an observation scale for VSN in ADL [40, 41] and will be filled out by a therapist or proxy (partner or caregiver). Neglect severity will be scored for each of 10 items, resulting in a total score of 0 (no neglect) to 30 (severe neglect).

##### Subjective neglect questionnaire (SNQ)

The SNQ is a 19-item questionnaire that will be administered to patients and proxies, asking them to rate the presence of common problems associated with neglect [42]. Each item will be scored on a five-point scale according to the frequency of the occurrence of the difficulty (ranging from at most once a month to at least once a day). The minimum score of 19 indicates no reported problems, the maximum score is 95 [18].

#### Baseline descriptors

The following data will be collected: demographics (age, gender, handedness, educational level), stroke characteristics (time post-stroke, lesion side, stroke type (ischemic, intracerebral or subarachnoid haemorrhage) and stroke history (first-ever or recurrent)), and global cognitive functioning (Montreal Cognitive Assessment; MoCA version 8.1 [43]).

### Randomization, blinding and treatment allocation

Participants will be randomly assigned to either the active tACS plus VST group *or* the sham tACS plus VST group. We will apply minimization, a method of adaptive stratified sampling, to prevent imbalances of potential confounders between the active and sham group. This will be achieved using “MinimPy”, an open-source customizable minimization program for allocation of patients to parallel groups in clinical trials [44]. Patients will be stratified according to the following factors: age (18-59/60-80), gender (male/female), and having had previous neglect treatment (yes/no). The software will automatically send an e-mail with the randomization results to a not-closely involved and only un-blinded research assistant. This assistant will then pick a 5-digit code from the list of codes provided in the NeuroConn DC Stimulator user manual (neuroConn GMBH) that either initiates the preprogrammed active stimulation protocol or the sham protocol. The un-blinded assistant will assign this unique code to the enrolled patient in question and will send the code to the blinded researchers. The un-blinded assistant will further not be involved in the study, so will play no further role in inclusion, testing or analyses. The blinded researchers will perform the intervention and administer the outcome measurements, independently of the un-blinded assistant.

Patients will also be blinded to treatment allocation.

### Intervention

The intervention (VST with active or sham ACS) will be offered by the researchers at the patients’ homes. In every training session, the patients will perform the VST on a touch screen laptop (HP EliteBook × 360 1040 G5 Notebook; screen size: 14 in.), whilst also receiving the (active or sham) stimulation. The VST lasts as long as the stimulation is applied (40 min). In total, patients will receive 18 training sessions in 6 weeks (3 sessions per week).

#### VST

All patient will receive computerized VST. The aim of the conventional VST is to train VSN patients to actively explore and consciously pay attention to stimuli on the contralesional side [16]. The conventional VST is similar to our digitized version. Patients’ visual search is systematically guided by contralesional cues (e.g., a visual stimulus of reference on the left) and by the researcher’s feedback [16].

Our VST program consists of several digitalized, evidence-based training tasks: 1) digit detection; 2) copying of line drawings on a dot matrix; 3) figure description; 4) reading training (tasks 1-4 based on [16]); 5) fill-out objects (based on [45]); 6) figure search (based on cancellation tasks, e.g. [30, 46]); 7) congruent movement training [47]; and 8) eye-movement training (“standard VST” in [47]).

#### Active tACS

The experimental group will receive active tACS during each session of the VST. To understand how tACS can correct for the attentional bias seen in VSN patients, in the next paragraph we elaborate on the rationale of our study.

Previous electroencephalography studies with healthy participants have linked attention shifts to alpha power in posterior parietal cortices [23–26]. To specify, increased alpha power reflects suppression of incoming sensory information. Thus, shifting attention to the right hemifield is accompanied by alpha power increases in the right hemisphere (inhibiting the unattended left hemifield) and alpha power decreases in the left hemisphere (release from inhibition). Interesting for the field of neglect rehabilitation is that previous studies have shown that tACS can increase the power of the alpha frequency [48, 49]. If indeed alpha power is increased in the ipsilateral relative to the contralateral side of attention, we hypothesize that the bias in visuospatial attention seen in neglect patients can be corrected for by boosting the alpha power in the contralesional parietal cortex by tACS.

Therefore, in the current study a small circular (diameter: 2.1 cm, thickness: 2 mm) and a large (outer diameter: 11 cm; inner diameter: 9 cm, thickness: 2 mm) rubber ring tACS electrode (NeuroConn, Ilmenau, Germany) will be placed onto the contralesional parietal cortex, with the small electrode positioned over P3 or P4 (based on the international 10-20 EEG system) and the large electrode centered around it. This ring electrode montage enables a higher spatial focality as compared to standard rectangular electrodes [50]. TACS ring electrodes will be attached to the patient’s head with conductive gel (ten20 paste, Weaver and Company, Aurora, CO, USA). The conductive gel will be used to reduce the impedance between skin and electrodes to below 10 kΩ.

Stimulation frequency and peak-to-peak intensity will be set to 10 Hz and 1.5 mA, phase offset will be set to 0 and 100 cycles (10s) will be used for ramping up. At the start of the VST, the tACS will be started. When the training is finished, after maximally 40 minutes, the tACS will be switched off.

#### Sham tACS

The placebo group will receive sham tACS, using the same device and electrodes positioned over the same location (P3 or P4), which is an inactive form of stimulation during which the patient believes he/she is being stimulated normally. We will implement sham tACS by ramping down the current immediately after the ramp up period. This way, the patient feels the ramp up and ramp down (which are the most noticeable in tCS), but does not receive a significant dose of tCS [51].

### Sample size estimates

To our knowledge, we are the first to combine an oscillatory-based transcranial brain stimulation protocol (10-Hz tACS) with conventional neglect treatment (VST). Other forms of NIBS (repetitive transcranial magnetic stimulation; rTMS, theta burst stimulation; TBS, transcranial direct current stimulation; tDCS) have indeed been used in combination with neglect treatment previously, but there, however, the rationale was to improve neglect based on conventional theoretical models, which prescribe ‘re-balancing’ activity between the hemispheres, via excitatory stimulation of the under-active injured hemisphere, or inhibition of the hyperactive intact hemisphere, or a combination of both. Since the current study is a conceptually novel approach, we estimated the necessary sample size based on the results of previous neglect studies that combined neglect treatment with NIBS that aimed at such ‘re-balancing’ in repeated sessions. The review of Van Lieshout and colleagues [52] reports four such RCT’s for which effect sizes are known or could be calculated. Cohen’s d ranged from 1.07-5.27 in two rTMS studies [53, 54], 1.48-7.14 in two TBS studies [54, 55], and 1.50-2.35 in one tDCS study [56]. In summary, all of the studies showed large effect sizes. Since these previous studies all took place in (sub-)acute patients, in which spontaneous neurological recovery can still occur [57], we choose an effect size of 0.8, which is lower than the previously reported range of effect sizes, but which is still commonly considered as a large effect size.

In our study, we will compare the CVDT test score before and after the six-week training period. To calculate the required sample size of our study population, we made use of G*Power (version 3.1) [58]. To find an effect size of d = .80 (Cohen’s f = .40), we calculated parameters for a repeated measures ANOVA with a 2 × 2 design (within-between interaction, 2 groups, and 2 testing sessions), a power of 0.80 and an alpha of .05, which yielded a total required sample size of 16 patients (8 per group). This is a conservative estimate, as mixed linear modeling, the method we will use to analyze our data, is more powerful than repeated measures ANOVA [59]. We choose to set this to 22 patients (11 per group) because patients may dropout due to the relatively long training period of 6 weeks (dropout estimated at 25%). The abovementioned RCT’s included a comparable number of patients per group (approximately 10 per group) and reported significant effects and large effect sizes, so we too expect that our design will have sufficient power, which we confirm with our a priori sample size calculation.

### Statistical analyses

The background characteristics of the patients will be described by using descriptive statistics. Baseline characteristics of the intervention and control group will be compared to detect differences at the start of the trial. Linear mixed model regression analyses with random effects for intercept and slope will be used to test for change in the primary and secondary outcome measures both within and between group. The predictors of theoretical interest are the effects of time and group and the interaction between time and group (fixed effects). Baseline score of the outcome measure, time since stroke, gender, and age will be introduced as potential fixed covariates. This is regardless whether or not these variables differ between groups, to enhance the fit of the model. Post-hoc contrasts will be performed for the interaction between time and group to test differences in treatment effects by intervention group allocation. The intention-to-treat principle is used by including all patients as randomized in the analyses, regardless of whether they received the complete program (dropout, non-adherence). Significance is set to *p* < .05 (two-sided). Analyses will be performed in IBM SPSS Statistics version 26.

### Data monitoring committee

No data monitoring committee was set up for this research.

## Discussion

One of the most important aspects of tACS is its ability to achieve cortical (brain activity) changes (even outlasting the stimulation) and to be able to put the brain in a state in which the effects of standard treatment can be bigger and/or longer-lasting. Modification of brain activity, by means of cortical stimulation, may improve the patient’s ability to relearn or acquire new strategies for carrying out a behavioral task, by facilitating local activity or by inhibiting maladaptive competing activity from other brain areas [60]. Previous studies have shown that NIBS during or before a learning process may yield behavioral improvements that are more robust and stable [61–63]. Highly relevant in the context of tACS, is that the effect of tACS depends on the state of the brain [64–66], and this state-dependence further offers currently unexplored options such as combining tACS with cognitive-behavioral interventions for synergistic augmentation.

Another strength of this study is that the use of digitized tests and training tasks will allow for a highly precise and detailed data collection, which opens the possibility to assess (subtle) progression on innovative outcome measures during training. Other strengths concern the study design (i.e. randomized and double-blind design), and range of outcome measures (i.e. ADL measures, follow-up assessments).

In the current study, the intervention (VST and active/sham tACS) will be offered by the research team. If proven effective, an exploration of the implementation of a tACS-VST rehabilitation program in chronic stroke care will be necessary, after which tACS-VST could be implemented as a treatment option for VSN.

## Data Availability

Data sharing is not applicable to this article as no datasets were generated or analyzed during the current study.

## Abbreviations

ADL: Activities of daily living
BB: Balloons-subtest B
BT: Bells task
BTT: Baking tray task
CBS: Catherine Bergego scale
CVDT: Computerized visual detection task
dPL: Left endpoint weighting
dPR: Right endpoint weighting
EWB: Endpoint weighting bias
MoCA: Montreal cognitive assessment
MLBT: McIntosh line bisection task
MLBT-d: McIntosh line bisection task-digitized
NIBS: Non-invasive brain stimulation
QoS: Quality of search
RCT: Randomized controlled trial
SCT: Star cancellation task
SLBT: Schenkenberg line bisection task
SNQ: Subjective neglect questionnaire
tACS: Transcranial alternating current stimulation
tES: Transcranial electrical stimulation
TMS: Transcranial magnetic stimulation
VSN: Visuospatial neglect
VST: Visual scanning training
